# Impedance cardiography (electrical velocimetry) and transthoracic echocardiography for non-invasive cardiac output monitoring in pediatric intensive care patients: a prospective single-center observational study

**DOI:** 10.1186/s13054-014-0603-0

**Published:** 2014-11-19

**Authors:** Martin Ernst Blohm, Denise Obrecht, Jana Hartwich, Goetz Christoph Mueller, Jan Felix Kersten, Jochen Weil, Dominique Singer

**Affiliations:** Department of Pediatrics, Division of Neonatology and Pediatric Intensive Care, University Medical Center Hamburg-Eppendorf, Martinistr. 52, Hamburg, 20246 Germany; Current address: Department of Neurology, Elbe Kliniken Stade, Bremervörder Str. 111, Stade, 21682 Germany; Department of Pediatric Cardiology, University Medical Center Hamburg-Eppendorf, Martinistr. 52, Hamburg, 20246 Germany; Department of Medical Biometry and Epidemiology, University Medical Center Hamburg-Eppendorf, Martinistr. 52, Hamburg, 20246 Germany

## Abstract

**Introduction:**

Electrical velocimetry (EV) is a type of impedance cardiography, and is a non-invasive and continuously applicable method of cardiac output monitoring. Transthoracic echocardiography (TTE) is non-invasive but discontinuous.

**Methods:**

We compared EV with TTE in pediatric intensive care patients in a prospective single-center observational study. Simultaneous, coupled, left ventricular stroke volume measurements were performed by EV using an Aesculon® monitor and TTE (either via trans-aortic valve flow velocity time integral [EVVTI], or via M-mode [EVMM]). H_0_: bias was less than 10% and the mean percentage error (MPE) was less than 30% in Bland–Altman analysis between EV and TTE. If appropriate, data were logarithmically transformed prior to Bland–Altman analysis.

**Results:**

A total of 72 patients (age: 2 days to 17 years; weight: 0.8 to 86 kg) were analyzed. Patients were divided into subgroups: organ transplantation (OTX, *n* =28), sepsis or organ failure (SEPSIS, *n* =16), neurological patients (NEURO, *n* =9), and preterm infants (PREM, *n* =26); Bias/MPE for EVVTI was 7.81%/26.16%. In the EVVTI subgroup analysis for OTX, NEURO, and SEPSIS, bias and MPE were within the limits of H_0_, whereas the PREM subgroup had a bias/MPE of 39.00%/46.27%. Bias/MPE for EVMM was 8.07%/37.26% where the OTX and NEURO subgroups were within the range of H_0_, but the PREM and SEPSIS subgroups were outside the range. Mechanical ventilation, non-invasive continuous positive airway pressure ventilation, body weight, and secondary abdominal closure were factors that significantly affected comparison of the methods.

**Conclusions:**

This study shows that EV is comparable with aortic flow-based TTE for pediatric patients.

## Introduction

In the 1960s, impedance cardiography was developed to monitor cardiac output (CO) [1]. This method is based on a change in resistance during the cardiac cycle to a transcutaneously applied electrical AC voltage, and is used to calculate left ventricular stroke volume (LVSV), and thus CO. After several modifications to the algorithm [1-5], impedance cardiography (that is, electrical velocimetry (EV)) devices have become commercially available. There is conflicting evidence on the use of EV in the literature [6-12], and the technique is not yet widely used clinically.

This study evaluated continuously applicable and non-invasive EV and compared it with discontinuously applicable and non-invasive transthoracic echocardiography (TTE). We compared LVSV measurements with EV and TTE in pediatric and neonatal patients, and analyzed parameters that affected comparison of the methods.

## Materials and methods

### Study design

This single-center observational study aimed to validate EV compared with TTE in pediatric intensive care patients with normal cardiac biventricular anatomy. LVSV was simultaneously measured by EV and TTE. Equivalence of EV and TTE was assumed if Bland–Altman analysis had bias <10% and mean percentage error (MPE) <30% regarding LVSV measurement by EV compared with TTE (H_0_) [13].

### Electrical velocimetry measurements

An Aesculon® monitor (CE 0123; Osypka Medical, Berlin, Germany) was used to record EV. The electrode position of four RedDot® neonatal ECG radiolucent prewired monitoring electrodes (3M Health Care, Neuss, Germany) was chosen as recommended by the manufacturer. The analyzed heart beats were recorded simultaneously by TTE on the Aesculon® monitor. The signal that was generated by the Aesculon® monitor for EV LVSV measurements was accepted if the green signal quality bar indicated a reliable signal.

### Transthoracic echocardiography measurements

For echocardiography, either the GE Medical Systems Vivid 7 (CE 0470; GE Healthcare, Munich, Germany) or the GE Healthcare Technologies Logiq P5 (CE 0459; GE Healthcare) ultrasound machine was used. LVSV by TTE was calculated using two different methods [14,15]. In one method, LVSV was calculated based on measurement of the flow velocity time integral (VTI) measured over the aortic valve (measured from an apical four-chamber view with angle correction, if necessary) multiplied by the area of the aortic valve:

$$ \mathrm{LVSV} = \mathrm{aortic}\ \mathrm{valve}\ \mathrm{area} \times \mathrm{V}\mathrm{T}\mathrm{I} $$

where the aortic valve diameter was determined by triplicate measurements of the internal diameter of the aortic valve hinge points:

$$ \mathrm{Aortic}\ \mathrm{valve}\ \mathrm{area} = {\left(0.5 \times \mathrm{diameter}\right)}^2 \times 3.14 $$

In the other method, LVSV was based on M-mode measurement in the long parasternal axis, using the internal algorithm of the echocardiography machine based on the Teichholz equation [16]. For these M-mode measurements, a single beat was simultaneously measured in triplicate by TTE and EV.

### Setting

Three consecutive heart beats for VTI measurements or a single beat for the M-mode measurement were simultaneously recorded with the corresponding identical EV beats. All of the TTE measurements were performed by a single operator (MEB).

### Patients and sample characteristics

Pediatric and neonatal patients treated at the University Medical Center Hamburg–Eppendorf (UKE) in the pediatric and neonatal ICUs were eligible. This study was approved by the ethics committee of the Chamber of Physicians Hamburg, Germany. This study was performed in accordance with the ethical standards laid down in the 1964 Declaration of Helsinki and its later amendments. National laws were observed. Parental written informed consent was obtained prior to data collection.

### Data collection and statistics

The distribution of data was graphically assessed. Right-skewed data were logarithmically transformed prior to statistical analysis. The intra-class correlation for repeated measurements on the same day in individual patients was high (97% for comparison of EV versus the LVSV measurement based on the VTI over the aortic valve (EVVTI), 98% for comparison of EV versus the LVSV determination by M-mode echocardiogram (EVMM)). Data were therefore combined into 285 measurement pairs (EV vs. TTE) by averaging the LVSV measured by each method in an individual patient, thus representing a single day of measurement in a single patient (*n* = 146 EVVTI combined data points, *n* = 139 EVMM combined data points). Agreement between the EV and TTE methods was assessed by means of Bland–Altman plots [17,18]. The MPE was computed as:

$$ \mathrm{M}\mathrm{P}\mathrm{E} = \mathrm{precision}\ /\ \left(\left({\mathrm{mean}}_{\mathrm{EV}} + {\mathrm{mean}}_{\mathrm{TTE}}\right)\ /\ 2\right) $$

where precision was defined as the two-standard-deviation method difference [13,17,19]. True precision for EV (TP_EV_) was calculated based on the following equation [20,21]:

$$ {\mathrm{TP}}_{\mathrm{EV}} = \surd\ \left({\left(\mathrm{M}\mathrm{P}\mathrm{E}\right)}^2\hbox{--}\ {\left({\mathrm{precision}}_{\mathrm{TTE}}\right)}^2\right) $$

A mixed-model analysis (analysis of covariance) was performed to identify the effects of persistent ductus arteriosus (PDA), persistent foramen ovale, the combination of PDA and persistent foramen ovale, non-invasive continuous positive airway pressure (CPAP) ventilation, any form of ventilation, use of catecholamines, outcome, body weight, secondary abdominal closure, sex, and heart rate on EV and TTE. *Post-hoc* power analysis was performed and the least significant change was calculated [20,22]:

$$ \mathrm{Minimum}\ \mathrm{change}\ \mathrm{t}\mathrm{hat}\ \mathrm{needs}\ \mathrm{t}\mathrm{o}\ \mathrm{b}\mathrm{e}\ \mathrm{measured}\ \mathrm{b}\mathrm{y}\ \mathrm{a}\ \mathrm{device}\ \mathrm{t}\mathrm{o}\ \mathrm{recognize}\ \mathrm{a}\ \mathrm{real}\ \mathrm{change} = \mathrm{precision} \times \surd 2 $$

The trend-following characteristic over repeated measurements was determined using Kendall's coefficient of concordance. The statistical significance level was set to 0.05. SPSS version 20.0 (IBM, Armonk, NY, USA) was used for all statistical analyses.

## Results

The study cohort included 72 pediatric intensive care patients (39 girls and 33 boys). The patients were classified into four subgroups: solid organ transplantation (OTX subgroup, *n* = 28: liver, *n* = 24; kidney, *n* = 2; combined liver and kidney transplant, *n* = 2), sepsis or other organ failure (SEPSIS subgroup, *n* = 16), acute neurological patients (NEURO subgroup, *n* = 9), and preterm infants (PREM subgroup, *n* = 26; gestational age 25 + 5 weeks to 34 + 5 weeks). Some patients (*n* = 7) could be classified into two subgroups (NEURO and SEPSIS, *n* = 5; OTX and SEPSIS, *n* = 2). In these 72 patients, 855 paired measurements of LVSV by EV and TTE (EVVTI, 438 paired measurements; EVMM, 417 paired measurements) were recorded with two sets of measurements on two separate days in most cases. In some patients, up to six sets of measurements were taken on separate occasions during their stay in the ICU. As described above, data were combined into 146 EVVTI data points and 139 EVMM data points, each representing a pair of measurements of echocardiography versus impedance cardiography on a single day in an individual patient. Table 1 presents detailed information on the patient and sample characteristics.

**Table 1:** **Patient and sample characteristics of electrical velocimetry versus transthoracic echocardiography, based on 855 paired measurements**

**Characteristic**	**Total**	**OTX**	**SEPSIS**	**NEURO**	**PREM**
**Patients in each group (** ***n*** **)**	72	28	16	9	26
**Paired measurements (** ***n*** **)**	855	372	213	96	243
**Number of measurement days per patient (mean)**	1.98	2.20	2.05	1.56	1.60
**Weight (kg) (median/mean, range)**	7.2/15.96 (0.84 to 86.00)	10.0/18.64 (2.06 to 86.00)	23.0/26.23 (2.06 to 60.00)	14.5/18.90 (3.10 to 45.00)	1.67/1.66 (0.84 to 2.40)
**Age (years) (median/mean, range)**	0.78/4.19 (0.01 to 17.87)	1.56/4.76 (0.05 to 17.87)	7.10/7.66 (0.16 to 17.87)	2.89/4.16 (0.04 to 11.12)	0.03/0.04 (0.01 to 0.11)
**Use of inotropes (%)**	29.47	37.27	50.47	50.00	0.00
**Mechanical ventilation via endotracheal intubation (%)**	39.30	46.11	51.89	93.75	4.94
**Any form of respiratory support (that is, non-invasive CPAP or ET tube) (%)**	51.58	49.33	54.72	93.75	43.21
**Secondary closure of the abdomen (%)**	7.37	16.94	2.83	0.00	0.00
**ICU survival rate (%)**	85.61	91.96	75.94	50.00	100.00

Data are shown for the whole pediatric cohort and for subgroups. CPAP, continuous positive airway pressure; ET, endotracheal; NEURO, acute neurological patients subgroup; OTX, solid organ transplantation subgroup; PREM, preterm infants subgroup; SEPSIS, sepsis or other organ failure subgroup.

The mean cardiac index was 4.0 l/(minute*m^2^) by EV, compared with 4.2 l/(minute*m^2^) by LVSV measurement based on the VTI over the aortic valve and with 4.5 l/(minute*m^2^) by LVSV determination by M-mode echocardiogram. Because the study included two separate TTE methods compared with EV (based on transaortic flow measurement (EVVTI) and M-mode measurement (EVMM)) the results are presented separately for these two study arms. There was a highly significant (*P* <0.001) positive Pearson correlation of LVSV measurements by EV and TTE for the whole cohort (EVVTI, r *=* 0.934; EVMM, *r* = 0.896) and for subgroup analyses for OTX subgroup (EVVTI, *r* = 0.929; EVMM, *r* = 0.869), SEPSIS subgroup (EVVTI, *r* = 0.911; EVMM, *r* = 0.863) NEURO subgroup (EVVTI, *r* = 0.955; EVMM, *r* = 0.899), and PREM subgroup (EVVTI, *r* = 0.786; EVMM, *r* = 0.728).

**Figure 1:**
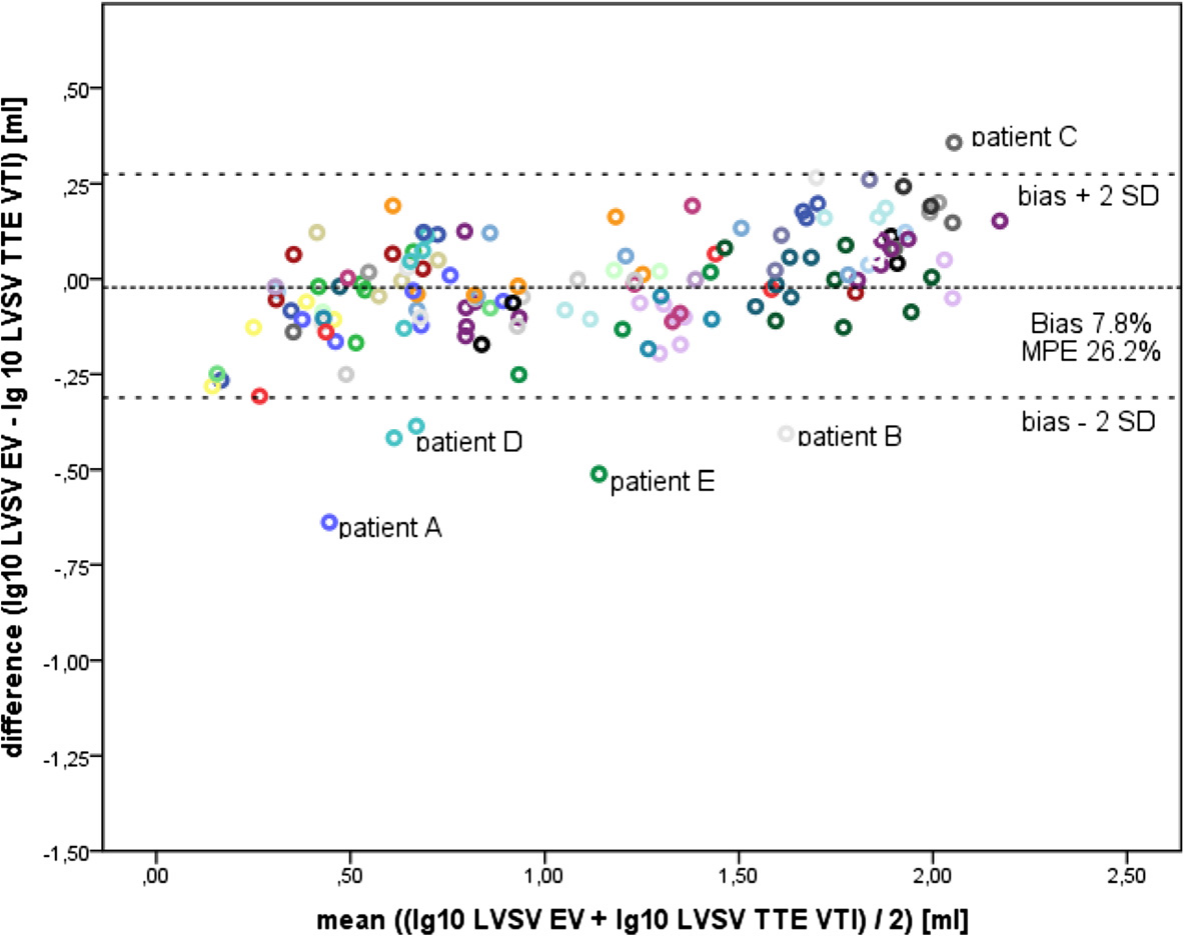
**Bland–Altman plot for comparison of left ventricular stroke volume measured by electrical velocimetry versus the transthoracic echocardiography velocity time integral.** Each data point represents combined paired measurements in an individual patient that were measured on separate occasions (146 combined paired measurements, logarithmic scale). Bias and two standard deviations (2SD) are shown as reference lines. EV, electrical velocimetry; LVSV, left ventricular stroke volume; MPE, mean percentage error; TTE, transthoracic echocardiography; VTI, velocity time integral.

**Figure 2:**
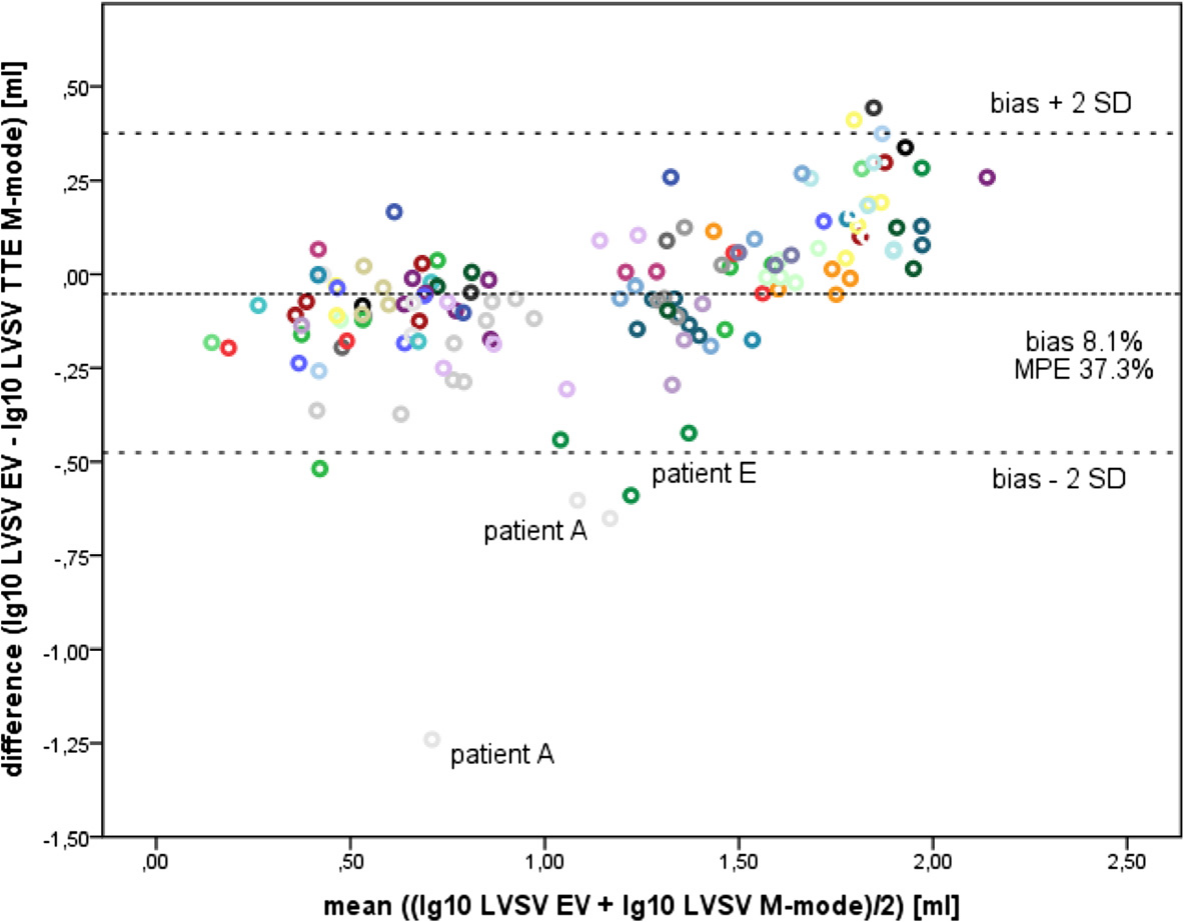
**Bland–Altman plot for comparison of left ventricular stroke volume measured by electrical velocimetry versus the transthoracic echocardiography M-mode.** Each data point represents combined paired measurements in an individual patient measured on separate occasions (139 combined paired measurements, logarithmic scale). Bias and two standard deviations (2SD) are shown as reference lines. EV, electrical velocimetry; LVSV, left ventricular stroke volume; MPE, mean percentage error; TTE, transthoracic echocardiography.

Bland–Altman plots for EVVTI and EVMM are shown in Figures 1 and 2. Outlying data points were not excluded from the Bland–Altman analysis. Reasons for the outliers are mentioned in Discussion. Bias and MPE are presented in Table 2. For EVVTI, the whole cohort and all subgroups (except for PREM subgroup) were within the cutoff levels of H_0_. For EVMM, data were within the limits of H_0_ only for OTX and NEURO subgroups, and outside the limits of H_0_ for the whole cohort and the other subgroups.

**Table 2:** **Comparison of measurement of left ventricular stroke volume by electrical velocimetry versus transthoracic echocardiography using Bland–Altman analysis**

**Study arm**	**Group**	**Number of patients/ measurements**	**Bias (%)**	**MPE (%)**
EVVTI	All	72/146	7.81	26.16
	OTX	28/65	5.05	23.03
	SEPSIS	16/36	2.62	26.82
	NEURO	9/16	6.98	14.79
	PREM	26/41	39.00	46.27
EVMM	All	72/139	8.07	37.26
	OTX	28/59	5.04	28.96
	SEPSIS	16/35	2.57	43.53
	NEURO	9/16	6.65	27.10
	PREM	26/40	39.53	42.80

Data were analyzed for subgroups. EVMM, electrical velocimetry versus the transthoracic echocardiography M-mode; EVVTI, electrical velocimetry versus the transthoracic echocardiography velocity time integral; MPE, mean percentage error; NEURO, acute neurological patients subgroup; OTX, solid organ transplantation subgroup; PREM, preterm infants subgroup; SEPSIS, sepsis or other organ failure subgroup.

**Figure 3:**
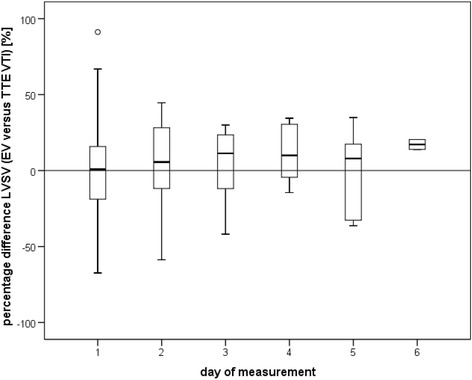
**Trend of left ventricular stroke volume measurements using electrical velocimetry versus measurements based on the velocity time integral over the aortic valve.** Percentage difference in repeated left ventricular stroke volume (LVSV) measurements between methods in individual patients on serial measurement days. Box plots show the median, standard deviation, and outliers (136 combined measurement points were included; outliers from Figure 1 were excluded). Note the constant average direction of bias over repeated measurement days. TTE, echocardiography; EV, electrical velocimetry; VTI, velocity time integral.

The trend of the relative differences in LVSV measurements over separate measurement occasions between the two methods is shown in Figure 3. There was no correlation between the relative differences and the times of individual measurements (*r* = 0.101, *P* = 0.24), and this was observed for the whole cohort and for combined data points with separate analyses for EVVTI and EVMM.

Kendall’s coefficient of concordance (to assess trend monitoring quality) did show a highly significant (*P* < 0.001) concordance of the relative bias of 97% for EVVTI and 100% for EVMM. Looking at the three repetitive measurements on individual measurement days, the concordance for separate measurement days was 96% for EVVTI and 89% for EVVM.

The average precision of TTE in transaortic flow measurements was 7.97% and that in M-mode measurements was 7.42% based on the calculation described by Cecconi and colleagues [20] with lower precision in the PREM subgroup (LVSV measurement based on the VTI over the aortic valve 29.55%/LVSV determination by M-mode echocardiogram 25.07%). TP_EV_ was 24.92% for EVVTI and 36.51% for EVMM. Subgroup analysis for EVVTI showed a better TP_EV_ for the more homogeneous patient subgroups OTX (22.22%) and NEURO (12.12%) compared with SEPSIS (26.75%) and PREM (35.60%). *Post-hoc* power analysis showed that the sample size was sufficient to detect a percentage difference in LVSV measurements of 9% for EVVTI and 12% for EVMM (at power = 0.8 and *P* = 0.05).

A mixed-model analysis was performed to identify parameters affecting any differences between EV and TTE (Table 3). A significant effect (for one or both of the comparisons EVVTI and EVMM) was observed for weight, secondary abdominal closure, endotracheal mechanical ventilation, and non-invasive nasal CPAP ventilation in the PREM subgroup. There tended to be an effect of sex on differences between EV and TTE (*P* = 0.062). Heart rate, outcome, and catecholamine use did not lead to a significant effect on differences between EV and TTE. In the PREM subgroup, CPAP ventilation (37% of the data points in the PREM subgroup were measured with CPAP) significantly (*P* = 0.022) affected EVVTI, but this effect was not observed for EVMM. For isolated PDA (5% of the data points for the PREM subgroup were with PDA) a trend was observed (*P* = 0.077 in EVVTI), but there was no effect of PDA in EVMM. There was no significant effect of a persistent foramen ovale in the PREM subgroup.

**Table 3:** **Possible parameters that affect differences in EVVTI and EVMM using mixed-model analysis**

**Parameter**	**Study arm**	**Effect on difference of methods (%)**	***P*** **value**
		**(95% limits of agreement)**	
Non-invasive CPAP in PREM	EVVTI	40.1 (34.6/46.4)	0.022
	EVMM	31.6 (26.6/37.6)	0.732
Ventilation via ET tube	EVVTI	5.0 (4.3/5.9)	0.017
	EVMM	4.7 (4.0/5.7)	0.069
Body weight	EVVTI	9.5 (6.4/7.5)	<0.001
	EVMM	11.5 (9.7/13.5)	<0.001
Secondary abdominal closure	EVVTI	5.2 (4.4/6.1)	0.272
	EVMM	6.9 (5.5/8.7)	0.002

CPAP, continuous positive airway pressure; ET, endotracheal; EVMM, electrical velocimetry versus the transthoracic echocardiography M-mode; EVVTI, electrical velocimetry versus the transthoracic echocardiography velocity time integral; PREM, preterm infants subgroup.

## Discussion

The main result of the study was good agreement for Bland–Altman analysis for EVVTI, with an acceptably low bias <10%, and MPE <30% [13] in the whole cohort and in the OTX, NEURO, and SEPSIS subgroups. However, bias and MPE in the PREM subgroup were above the accepted range. For EVMM, bias was within the range of H_0_ except in the PREM subgroup. For EVVM, MPE was above the range of H_0_ in the whole cohort and the PREM and SEPSIS subgroups (not OTX and NEURO) (Table 2).

A theoretical TP_EV_ could be calculated because two imprecise CO monitoring methods (EV and TTE) were compared [19-21]. In our study, this TP_EV_ was based on combined data points with triplicate measurements. TP_EV_ was better for the OTX and NEURO subgroups than for the less homogeneous subgroups SEPSIS and PREM. These findings are consistent with other validation studies on EV. Acceptable equivalence was reported [13] when comparing EV with other CO monitoring methods in homogeneous adult biventricular patients [9] and homogeneous pediatric patient groups [23,24]. Non-equivalence of EV compared with other methods (mostly due to a high MPE) has been reported in studies with homogeneous and heterogenic pediatric patient cohorts [21,25-29], with an effect of left-to-right shunts and ventricular septal defects on the compared methods [27], as well as in adult studies with heterogenic patient cohorts [11,12,30-32].

Transthoracic impedance is affected by air and fluid content in the chest. Changes in fluid content in the chest by pleural effusion, cardiac congestion, alterations in the size of cardiac chambers (for example, because of valvular insufficiency), or increased air content (for example, by application of CPAP or mechanical ventilation) affect basic impedance, and thus calculation of LVSV by EV. Changes in blood content or blood flow alter the size of the cardiac cycle-related change in impedance (for example, in cardiac valve insufficiency or in the presence of a relevant PDA). The outliers in the Bland–Altman plots in Figures 1 and 2 had one or several of the abovementioned factors limiting signal quality in EV (for example, Patient A had mitral endocarditis with mitral insufficiency (grade 4), Patient B was on high-frequency oscillation after bone marrow transplantation, and Patients C, D, and E all had organ transplant with abdominal patch closure or a large intraabdominal tumor prior to liver transplantation). These findings are consistent with a previous study showing a significant effect of extravascular lung water on impedance cardiography [33].

Mixed-model analysis was performed on parameters that theoretically affect the true LVSV and CO and/or impedance cardiography measurements. Parameters that affected differences between the methods of EV and TTE in this study were body weight, mechanical ventilation, and non-invasive CPAP ventilation. Inotropic support or outcome did not affect differences between EV and TTE. The parameter with the strongest effect was non-invasive CPAP ventilation, which contributed to 40.1% of the difference using EVVTI in the PREM subgroup. This result and our finding that PDA was present in only 5% of PREM measurements may have been the reason why PDA did not reach significance (*P* = 0.077) in the PREM group. A previous study compared CO by EV versus TTE in 28 preterm neonates, and showed that the difference between the methods was 4% for ventilated infants and 2.5% for nonventilated infants [23]. This previous finding is in accordance with the current study, which demonstrated an effect of ventilation on comparison of the methods.

### Theoretical limitations of the study

To achieve a simultaneously paired LVSV measurement of identical heart beats by EV and TTE, the echocardiography used in the present study was only in a single plane rather than in two opposing planes. The precision of TTE in this study might therefore not have been as good as 30%, which has been reported in the literature [19]. However, the precision of TTE might have been better than the MPE of 36% reported in children for systems with blind Doppler probe positioning [34,35]. The precision of TTE in this study was calculated as approximately 8%. Additionally, the precision of TTE was not homogeneous and depended on patient subgroups with lower precision, particularly in the PREM subgroup, contributing to higher bias and MPE in that subgroup.

Another limitation of the study may be due to variation in LVSV during the respiratory cycle. Our study used paired measurements irrespective of the respiratory cycle, and did not take into account that the variation in stroke volume was between 10 and 30% in most cases. In vigorously breathing neonates, the variation was as high as 40% (as indicated by the Aesculon® monitor). The variation in stroke volume during the respiratory cycle is a problem for any method of determining CO [26,30,36,37]. Theoretically, variation in stroke volume should equally affect EV and TTE.

Previous studies have shown a significant effect of extravascular lung water on impedance cardiography CO measurements based on the Sramek–Bernstein equation compared with CO by thermodilution [33]. This implies that heterogenic patients with different amounts of extravascular lung water (as in this study) have heterogenic EV measurements, and this will affect comparison of EV and TTE.

### Trend monitoring

To base goal-directed therapy on a monitoring tool such as EV CO measurement, the capacity of the applied method for determining a trend is important [20,38] (that is, the ability of the method to detect a change in CO over time). Figure 3, which shows repeated measurements over time, demonstrates that the average direction of bias between EV and TTE remained positive or negative with repeated measurements. The high Kendall’s concordance mathematically supports this visual estimate of the constancy of the bias both for repetitive tests on the same day and on different days. By increasing the sampling interval with more measurements or more heart beats, the MPE between the two compared methods will become less (that is, precision will improve) [20,22]. The least significant change is defined as the minimum change that needs to be measured by a device to recognize a real change [22]. According to the equation for calculating the least significant change, the minimum percentage of LVSV change detected by EV in this study was 11.3% (sampling interval of three heart beats). After five measurement cycles (equivalent to 15 heart beats), the least significant change for EV would theoretically [22] become 5%. This implies that at a heart rate of 60 beats/minute EV would theoretically be able to detect a 5% change in LVSV within 15 seconds of averaged measurement. This short time response to a relatively small change in LVSV implies good tracking properties of the EV method [38]. Other continuous CO monitoring devices, including non-invasive devices (for example, continuous Doppler-based systems) [34,35] and invasive devices (for example, transpulmonary thermodilution methods combined with pulse contour analysis) [11,12], normally average multiple cardiac cycles for more accurate continuous monitoring. Because EV is a non-invasive and continuously applicable method, combined measurement cycles averaging LVSV and CO over multiple heart beats are technically possible. This averaging would also equalize variation in stroke volume during the respiratory cycle. EV therefore appears to be suitable for monitoring trends. The advantages of non-invasiveness and continuity of EV, with a low bias compared with TTE, may outweigh the relatively low precision of this method.

In summary, LVSV measurements by EV and TTE were not different in pediatric intensive care patients if LVSV was determined by TTE using transaortic flow (EVVTI). This finding was observed for the whole cohort and for the subgroups OTX, NEURO, and SEPSIS, but not for the PREM subgroup. This equivalence could not be demonstrated because of a high MPE for EV and TTE if LVSV was determined by M-mode (EVMM), except in the OTX and NEURO subgroups. The poorer agreement between EV and TTE in the PREM subgroup may be related to the fact that nasal CPAP was the main parameter that affected differences between the methods. Because the average direction of bias between EV and TTE with repeated measurements in the same patients remained constant, EV appears to be a suitable method for following trends in CO.

## Conclusions

Because of partial or good agreement between the two methods EV and TTE for determining LVSV (depending on the group of patients), EV appears to be suitable for determination of CO in children. The advantage of continuous applicability theoretically facilitates the possibility of monitoring of CO trends by EV.

## Key messages

- In this study, stroke volume measurements based on EV and echocardiographic transaortic flow were not different in pediatric intensive care patients.
- In preterm infants, there was a difference between the two methods, but LVSV measurements were significantly correlated.
- The main factors that affected comparison between EV and echocardiography were any form of ventilation and body weight.
- EV appears to be suitable for monitoring trends in pediatric patients.

## Abbreviations

CO: cardiac output
CPAP: continuous positive airway pressure
EV: electrical velocimetry (impedance cardiography)
EVMM: comparison of electrical velocimetry versus the transthoracic echocardiography M-mode
EVVTI: comparison of electrical velocimetry versus the transthoracic echocardiography velocity time integral
LVSV: left ventricular stroke volume
MPE: mean percentage error
NEURO: neurological patient group
OTX: solid organ transplantation patient group
PDA: persistent ductus arteriosus
PREM: preterm infant patient group
SEPSIS: sepsis or organ failure patient group
TP_EV_: true precision for electrical velocimetry
TTE: transthoracic echocardiography
VTI: velocity time integral
